# Genomic Epidemiology of Global Carbapenemase-Producing *Enterobacter* spp., 2008–2014

**DOI:** 10.3201/eid2406.171648

**Published:** 2018-06

**Authors:** Gisele Peirano, Yasufumi Matsumura, Mark D. Adams, Patricia Bradford, Mary Motyl, Liang Chen, Barry N. Kreiswirth, Johann D.D. Pitout

**Affiliations:** University of Calgary, Calgary, Alberta, Canada (G. Peirano, J.D.D. Pitout);; Kyoto University Graduate School of Medicine, Kyoto, Japan (Y. Matsumura);; J. Craig Venter Institute, La Jolla, California, USA (M.D. Adams);; AstraZeneca Pharmaceuticals LP, Waltham, Massachusetts, USA (P. Bradford);; Merck & Co., Inc., Rahway, New Jersey, USA (M. Motyl);; Rutgers University, Newark, New Jersey, USA (L. Chen, B.N. Kreiswirth);; University of Pretoria, Pretoria, South Africa (J.D.D. Pitout)

**Keywords:** surveillance, Enterobacter spp., Enterobacteriaceae, carbapenemases, genomic epidemiology, antimicrobial resistance, bacteria

## Abstract

We performed whole-genome sequencing on 170 clinical carbapenemase-producing *Enterobacter* spp. isolates collected globally during 2008–2014. The most common carbapenemase was VIM, followed by New Delhi metallo-β-lactamase (NDM), *Klebsiella pneumoniae* carbapenemase, oxacillin 48, and IMP. The isolates were of predominantly 2 species (*E. xiangfangensis* and *E. hormaechei* subsp. *steigerwaltii*) and 4 global clones (sequence type [ST] 114, ST93, ST90, and ST78) with different clades within ST114 and ST90. Particular genetic structures surrounding carbapenemase genes were circulating locally in various institutions within the same or between different STs in Greece, Guatemala, Italy, Spain, Serbia, and Vietnam. We found a common NDM genetic structure (NDM-GE-U.S.), previously described on pNDM-U.S. from *Klebsiella pneumoniae* ATCC BAA-214, in 14 different clones obtained from 6 countries spanning 4 continents. Our study highlights the importance of surveillance programs using whole-genome sequencing in providing insight into the molecular epidemiology of carbapenemase-producing *Enterobacter* spp.

The emergence of carbapenem resistance is a major public health concern because these agents are regarded as one of the last effective therapies available for treating serious infections caused by *Enterobacteriaceae* (*1*). Carbapenemases are important causes of carbapenem resistance because they can be transferred between members of the *Enterobacteriaceae*. The most common carbapenemases among clinical *Enterobacteriaceae* are the *Klebsiella pneumoniae* carbapenemases (KPCs) (Amber class A), IMPs, VIMs, New Delhi metallo-β-lactamase (NDMs) (class B or metallo-β-lactamases), and oxacillin (OXA) 48–like (class D) enzymes (*2*).

Recent surveillance studies have shown that *Enterobacter* spp. are often the second or third most common *Enterobacteriaceae* species associated with carbapenemases (*3*,*4*). Typically, KPCs are common among *Enterobacter* spp. from the United States and South America (*5*). VIMs are often limited to Europe, NDMs to the Indian subcontinent, and OXA-48 to North Africa and the Middle East (*5*).

Comprehensive global data regarding the different *Enterobacter* species and molecular epidemiology are currently limited. We designed a study that used short-read whole-genome sequencing to describe the molecular characteristics and international distribution of *Enterobacter* spp. with different carbapenemases (n = 170) obtained from 2 global surveillance systems during 2008–2014.

## Materials and Methods

### Bacterial Isolates

We included 170 clinical, nonrepeat *Enterobacter* spp. collected from 2 global surveillance programs, namely the Merck Study for Monitoring Antimicrobial Resistance Trends (SMART) (2008–2014) and the AstraZeneca global surveillance program (2012–2014), presently known as the INFORM Global Surveillance Study of Antimicrobial Resistance (Technical Appendix 1 Table 1; Technical Appendix 2). The isolates initially underwent phenotypic identification and microdilution panel susceptibility testing, and all carbapenem-nonsusceptible isolates underwent molecular screening for *bla*_KPC_, *bla*_VIM_, *bla*_NDM_, *bla*_OXA-48_–like, *bla*_IMP_, and *bla*_GES_ as described previously (*6*). We obtained a total of 142,226 *Enterobacteriaceae* from the period 2008–2014, and 6,457 (4.5%) were identified as *Enterobacter* spp.; 682 were nonsusceptible to 1 of the carbapenems, and 170/6,457 (2.6%) were positive for *bla*_KPC_, *bla*_OXA-48_–like, *bla*_NDM_, *bla*_VIM,_ and *bla*_IMP_ and thus included in our study.

### Whole-Genome Sequencing

We used the Nextera XT DNA sample preparation kit (Illumina, San Diego, CA, USA) to prepare libraries for sequencing. We multiplexed and sequenced samples on an Illumina NextSeq500 for 300 cycles (151 bp paired-end).

### Genomic Analysis

We obtained draft genomes by using SPAdes version 3.10.1 (*7*). We identified species based on the *hsp60* gene sequences (*8*). We created whole-genome phylogenetic trees, including reference strains for identification of *E. cloacae* complex (*9*; Technical Appendix 1 Table 2).

To define the presence of genes and their alleles, we accessed BLAST in combination with the following databases or typing schemes: BLAST (http://blast.ncbi.nlm.nih.gov/Blast), National Center for Biotechnology Information (NCBI) Beta-Lactamase Data Resources (http://www.ncbi.nlm.nih.gov/pathogens/beta-lactamase-data-resources), ResFinder (*10*), PlasmidFinder (*11*), and *Enterobacter cloacae* Multilocus Sequence Typing (MLST) Databases (http://pubmlst.org/ecloacae). We classified integrons according to INTEGRALL (http://integrall.bio.ua.pt).

### Phylogenetic Analysis

We created a recombination-free, core single-nucleotide polymorphism (SNP)–based phylogenetic tree and identified SNPs by mapping the reads or aligning the genomes against *E. xiangfangensis* type strain LMG27195 (*9*) using the RedDog pipeline (https://github.com/katholt/RedDog). We removed recombination sites according to Gubbins (*12*) and removed prophages identified by PHAST (*13*). We included core SNPs and sites that were present in all genomes to create a maximum-likelihood tree using RAxML with the general time-reversible plus gamma substitution model (*14*). We visualized the tree by using iTOL version 3 (*15*).

To identify clades within certain sequence types (STs), we used a phylogeny-free population genetics approach of core SNPs, conducting hierarchal clustering analysis with the Bayesian Analysis of Population Structure program (*16*). We included all 1,048 available *Enterobacter* spp. genomes in the NCBI Reference Sequence Database (http://www.ncbi.nlm.nih.gov/refseq) as of June 20, 2017. An in silico MLST analysis identified 282 STs from 950 typeable genomes. We included a total of 201 genomes of STs 78, 90, 93, 105, 108, 114, and 171 for the clustering analysis (Technical Appendix 1 Table 3). For each *E. hormaechei* subspecies or *E. xiangfangensis*, the hierarchal Bayesian Analysis of Population Structure clustering analysis (*16*) was conducted with 3 nested levels with a priori upper bound of the number of clusters between one fourth to one half of the total number of isolates. We defined clades by using the second level of clustering.

### Sequence Data Accession Numbers

We deposited the sequencing data in the DNA Data Bank of Japan and NCBI (NCBI BioProjects PRJNA259658 and PRJNA398291) databases (accession nos. DRA004879, SRP046977, and SRR2960053–SRR2960159 [SMART isolates] and SRR5939895–SRR5939952 [AstraZeneca isolates]). The sequences of new integrons or genetic environments described in this study were GenBank accession nos. LC224310–2, MF288916–351991, and MF327263–71.

## Results

### Global Distribution of Carbapenemases among *Enterobacter *spp*.*

We included a total of 170 carbapenemase-producing *Enterobacter* strains in the study. The VIMs (VIM-1, 4, 5, 23, and 31; n = 51 [46 were only positive for VIM, and 5 co-produced OXA-48]) were the most common carbapenemase among this collection, followed by NDMs (NDM-1, 6, and 7; n = 43 [41 were positive only for NDM, 1 also co-produced OXA-48. and 1 co-produced KPC-2]); KPCs (KPC-2, 3, 4, and 5; n = 38 [37 were only positive for KPC, and 1 co-produced NDM]); OXA-48 (n = 31 [25 were only positive for OXA-48, 5 co-produced VIM, and 1 co-produced NDM]); and IMPs (IMP-1, 4, 8, 13, and 14; n = 14). *Enterobacter* spp. with *bla*_VIM_ were mostly limited to Europe; isolates with *bla*_NDM_ were present predominantly in the Balkans, India, and Vietnam; isolates with *bla*_KPC_ were mainly found in the United States and South America; isolates with *bla*_OXA-48_ were largely present in North Africa and the Middle East; and isolates with *bla*_IMP_ occurred mostly in the Philippines, Taiwan, and Australia. The global distribution of isolates from this study was similar to what had previously been reported for other members of *Enterobacteriaceae*, especially *Klebsiella* spp. with carbapenemases (*5*,*17*).

### *E. aerogenes* Distant from *E. cloacae* Complex

We identified 10 isolates as *E. aerogenes*. These results are described in Technical Appendix 2.

### *E. xiangfangensis* Identified as the Most Common Species

**Figure 1:**
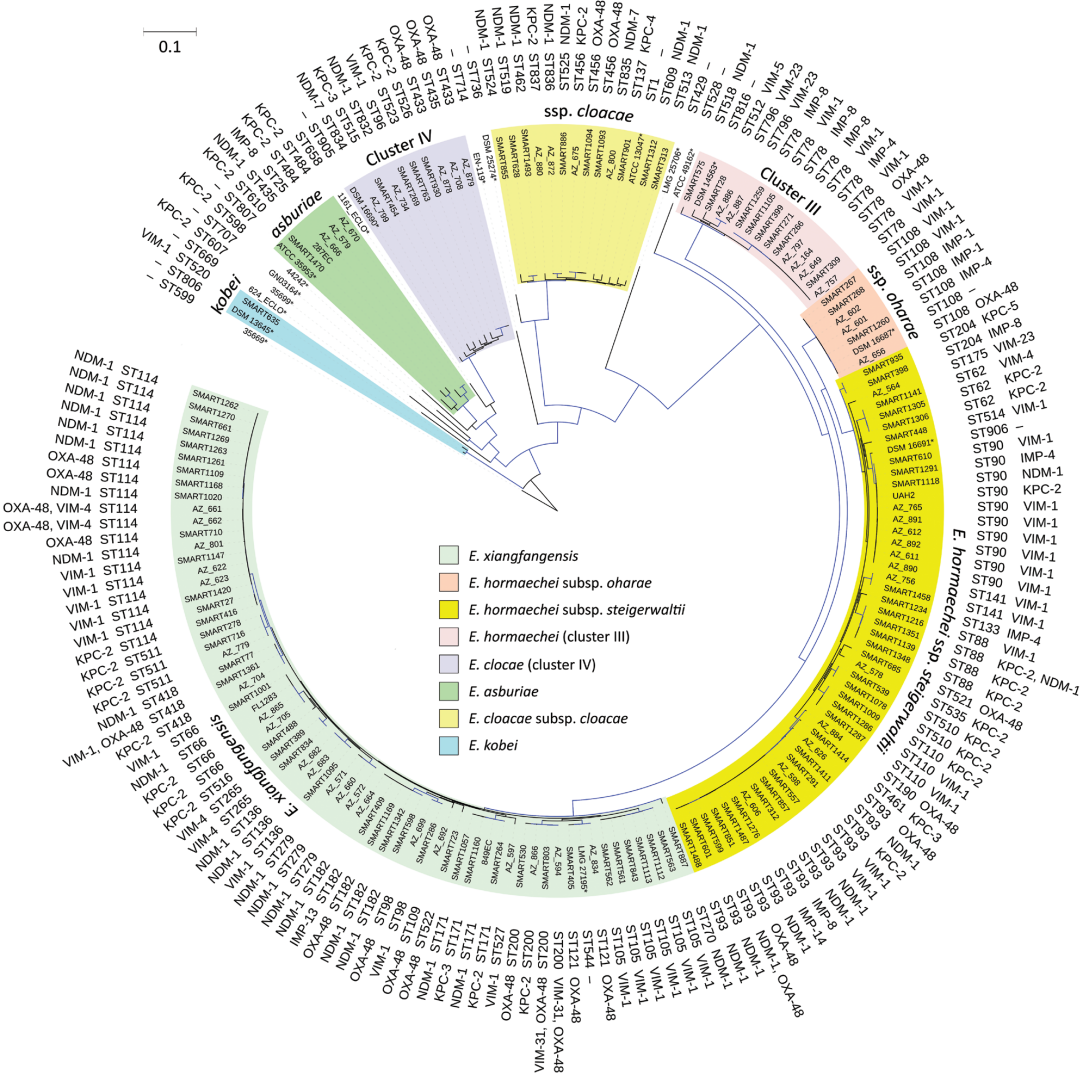
Phylogenetic tree of the different species and sequence types among 160 *Enterobacter cloacae* complex isolates identified from *Enterobacter* spp. isolates collected in the Merck Study for Monitoring Antimicrobial Resistance Trends, 2008–2014, and the AstraZeneca global surveillance program, 2012–2014. The tree is rooted with *E. cloacae* complex Hoffmann cluster IX (Chavda group R) strain 35,699. A total of 369,123 core single-nucleotide polymorphisms were found; 4,010 were used to draw the tree (after phages and recombination sites were excluded). KPC, *Klebsiella pneumoniae* carbapenemase; NDM, New Delhi metallo-β-lactamase; OXA, oxacillin; ST, sequence type; –, information missing; *, isolate identified in another study. Scale bar indicates nucleotide substitutions per site.

The *E. cloacae* complex (n = 160) from our study was obtained from intraabdominal (n = 69), urine (n = 56), skin and soft tissue (n = 19), blood (n = 2), and respiratory specimens (n = 14). We identified 8 species among *E. cloacae* complex (*E. xiangfangensis* [n = 65], *E. hormaechei* subsp. *steigerwaltii* [n = 47], *E. cloacae* cluster III [n = 14], *E. cloacae* subsp. *cloacae* [n = 13], *E. cloacae* cluster IV [n = 9], *E. hormaechei* subsp. *oharae* [n = 6], *Enterobacter asburiae* [n = 5], and *Enterobacter kobei* [n = 1]). These species were associated with different types of carbapenemases and showed global distribution (Figure 1; Technical Appendix 2 Table 1). *E. xiangfangensis* was frequent in the Balkans (e.g., Croatia, Romania, and Serbia), whereas *E. hormaechei* subsp. *steigerwaltii* was mostly prevalent in Greece and Vietnam (Technical Appendix 2 Table 2 Table 1). This overrepresentation was attributable to the presence of particular STs among these species (Technical Appendix 2 Table 2).

### Dominant Sequence Types Identified among 4 Species in *E. cloacae* Complex 

*E. xiangfangensis* from our study comprised 18 different STs, including 1 dominant ST, ST114 (19/65; 29%). *E. hormaechei* subsp. *steigerwaltii* comprised 15 different STs, including 2 dominant STs, ST90 (10/47; 21%) and ST93 (14/47; 30%). *E. cloacae* cluster III comprised 4 different STs, including 1 dominant ST, ST78 (10/14 [71%). All 6 of the *E. hormaechei* subsp. *oharae* isolates belonged to ST108 (Figure 1). The remaining species did not contain a dominant ST, and we found new STs among *E. cloacae* cluster IV (ST832 and ST834) and *E. cloacae* subsp. *cloacae* (ST835, ST836, and ST837).

### Major and Minor Sequence Types among *Enterobacter cloacae* Complex

Among the *E. cloacae* complex, we identified 4 major STs (>10 isolates/ST), ST114, ST93, ST90, and ST78. We also identified 2 minor STs (5–9 isolates/ST), ST105 and ST108.

**Figure 2:**
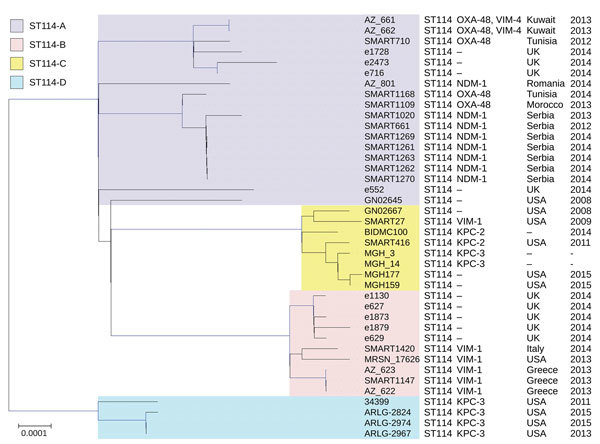
Phylogenetic tree of the different clades among 40 *Enterobacter xiangfangensis* ST14 isolates identified from *Enterobacter* spp. isolates collected in the Merck Study for Monitoring Antimicrobial Resistance Trends, 2008–2014, and the AstraZeneca global surveillance program, 2012–2014. The tree is rooted with *E. hormaechei* subsp. *hormaechei* isolate ATCC49162. A total of 317,867 core single-nucleotide polymorphisms were found; 27,705 were used to draw the tree (after phages and recombination sites were excluded). The isolates from other studies were negative for carbapenemases. KPC, *Klebsiella pneumoniae* carbapenemase; NDM, New Delhi metallo-β-lactamase; OXA, oxacillin; ST, sequence type; –, information missing. Scale bar indicates nucleotide substitutions per site.

ST114 (n = 19) from *E. xiangfangensis* was the most common ST and divided into 4 clades. Isolates representing 3 of the clades (ST114A, ST114B, and ST114C) were from this study, and isolates representing clade ST114D were from a different study (*9*; Figure 2; Technical Appendix 2 Table 2). ST114 had a global distribution (Greece, Italy, Kuwait, Morocco, Romania, Serbia, Tunisia, and the United States) and was associated with different carbapenemases (VIM-1, VIM-4+OXA-48, NDM-1, KPC-2, and OXA-48) (Technical Appendix 2 Table 2). The largest clade (ST114A [n = 13]) was present in Serbia, Romania (with *bla*_NDM-1_), Tunisia, Morocco, and Kuwait (with *bla*_OXA-48_) (Technical Appendix 2 Table 2). Clade ST114B (n = 4) with *bla*_VIM-1_ was obtained from Greece and Italy, and clade ST114C (n = 2; 1 with *bla*_VIM-1_ and 1 with *bla*_KPC-2_) was found in the United States. ST114 is a common global human *Enterobacter* clone (*18*) and is also present in companion animals (*19*). This international clone is associated with various antimicrobial resistance determinants (*20*) and was responsible for a prolonged nosocomial outbreak involving KPC-3 in the United States (*21*).

**Figure 3:**
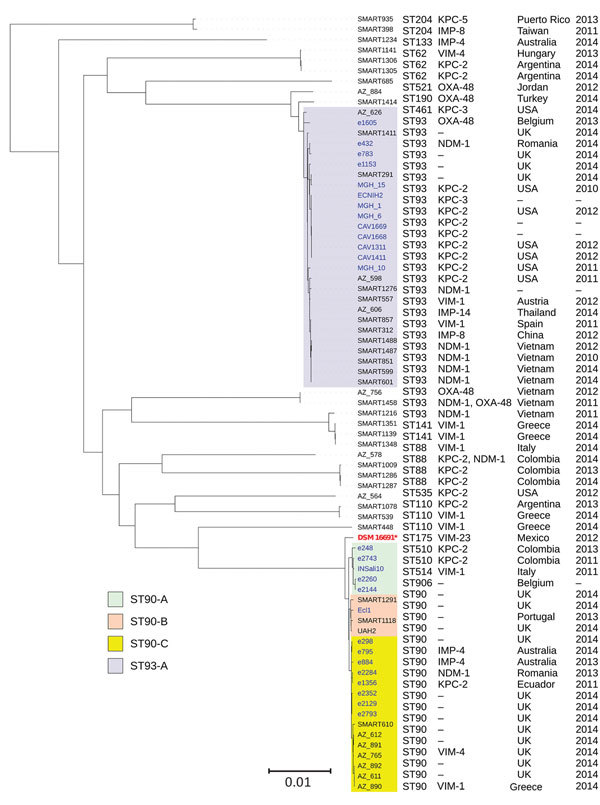
Phylogenetic tree of the different clades among 51 *Enterobacter hormaechei* subsp. *steigerwaltii* ST90 and ST93 isolates identified from *Enterobacter* spp. isolates collected in the Merck Study for Monitoring Antimicrobial Resistance Trends, 2008–2014, and the AstraZeneca global surveillance program, 2012–2014. The tree is rooted with *E. hormaechei* subsp. *hormaechei* isolate ATCC49162. A total of 317,867 core single-nucleotide polymorphisms were found; 27,705 were used to draw the tree (after phages and recombination sites were excluded). The isolates from other studies were negative for carbapenemases. Clades are grouped by color. KPC, *Klebsiella pneumoniae* carbapenemase; NDM, New Delhi metallo-β-lactamase; OXA, oxacillin; ST, sequence type; –, information missing. Scale bar indicates nucleotide substitutions per site.

ST93 (n = 14), from *E. hormaechei* subsp. *steigerwaltii*, was the second most common ST in this collection and consisted of 1 clade (Figure 3; Technical Appendix 2 Table 2). ST93 had a global distribution (Australia, Belgium, China, Romania, Spain, Thailand, United States, and Vietnam) and was associated with different carbapenemases (IMP-8, IMP-14, VIM-1, NDM-1, KPC-2, and OXA-48). ST93 was mostly present in Vietnam (n = 7), where it contained *bla*_NDM-1_ and *bla*_OXA-48_ (Technical Appendix 2 Table 2).

ST90 (n = 10), from *E. hormaechei* subsp. *steigerwaltii,* and ST78 (n = 10), from *E. cloacae* cluster III, were the next most common STs in our collection. ST90 was divided into 3 clades; isolates from 2 of the clades (ST90B and ST90C) were from this collection, whereas isolates representing clade ST90A were from a different study (*22*; Figure 3; Technical Appendix 2 Table 2). ST90C with *bla*_VIM-1_ (n = 7) was from Greece, whereas clade ST90B showed an international distribution (ST90C with IMP-4 from Australia, KPC-2 from Canada, and NDM-1 from Romania).

ST78 from *E. cloacae* cluster III consisted of 1 clade. This ST was associated with VIM-1 (Greece, Italy, and Spain), IMP-4 (Philippines), IMP-8 (Taiwan), and OXA-48 (Turkey) (Technical Appendix 2 Table 2).

**Figure 4:**
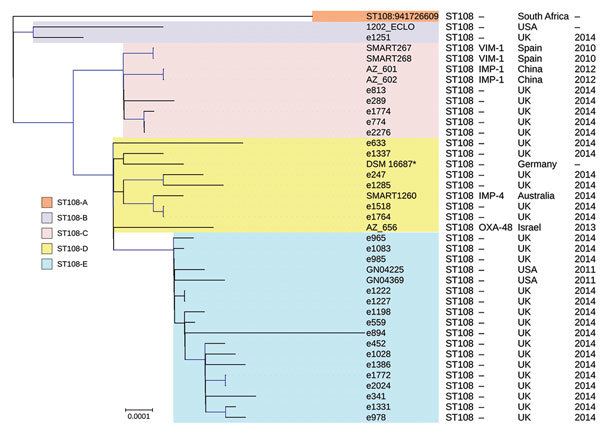
Phylogenetic tree of the different clades among 39 *Enterobacter hormaechei* subsp. *oharae* ST108 isolates identified from *Enterobacter* spp. isolates collected in the Merck Study for Monitoring Antimicrobial Resistance Trends, 2008–2014, and the AstraZeneca global surveillance program, 2012–2014. The tree was rooted with *E. hormaechei* subsp. *hormaechei* isolate ATCC49162. A total of 317,867 core single-nucleotide polymorphisms were found; 27,705 were used to draw the tree (after phages and recombination sites were excluded). The isolates from other studies were negative for carbapenemases. Clades are grouped by color. KPC, *Klebsiella pneumoniae* carbapenemase; NDM, New Delhi metallo-β-lactamase; OXA, oxacillin; ST, sequence type; –, information missing. Scale bar indicates nucleotide substitutions per site.

The minor STs, including ST105 and ST108 (both with 6 isolates), were distinguished on the basis of their molecular epidemiology. ST105 from *E. xiangfangensis* belonged to a single clade and was only present in Croatia, where it contained *bla*_VIM-1_. All the *E. hormaechei* subsp. *oharae* isolates belonged to ST108, which was divided into 5 clades; isolates from 2 of the clades (ST108C and ST108D) were from this collection, whereas isolates representing the other clades were from different studies (*23*; Figure 4). Clade 108C (n = 4) was present in Spain with *bla*_VIM-1_ (n = 2) and China with *bla*_IMP-1_ (n = 2), and ST108D (n = 2) was found in Australia (with *bla*_IMP-4_) and Israel (with *bla*_OXA-48_).

### β-lactamases, Antimicrobial Resistance Determinants, and Plasmid Analysis

For each of the 170 isolates, we tabulated the study number, GenBank accession number, species, date, country of isolation, ST, and clade. The β-lactamases, antimicrobial resistance determinants, plasmid replicon types, and plasmid STs are shown in Technical Appendix 1 Table 1 and Technical Appendix 2.

### Genetic Environments Surrounding the Carbapenemase Genes

We were able to successfully characterize the immediate genetic environments surrounding the carbapenemase genes in 8/14 *E. cloacae* complex with IMP, 28/33 with KPC (including 4 novel structures named KPC-GE01, KPC-GE02, KPC-GE03, and KPC-GE04), 42/42 with NDM (including 4 novel structures named NDM-GE01, NDM-GE02, NDM-GE03, and NDM-GE04), 17/27 with OXA-48 (including 4 novel structures named OXA-GE01, OXA-GE02, OXA-GE03, and OXA-GE04), and 46/51 with VIM (including the novel integrons In*1372*, In*1373*, and In*1374*) (Technical Appendix 2 Table 3). We have also described the novel structures found in *E. aerogenes* (Technical Appendix 2).

The *bla*_KPC_ were mainly associated with the Tn*4401b* isoform (including the 4 novel structures), whereas *bla*_OXA-48_ was always associated with Tn*1999* (including the 4 novel structures). Isolates with NDM contained IS*Aba125* upstream and *ble*_MBL_ downstream of the *bla*_NDM_, and the *bla*_VIM_ and *bla*_IMP_ were situated within diverse class I integrons from various countries (Technical Appendix 2 Table 2).

### Integrons Harboring *bla*_VIM-1_ Circulating Locally within the Same or between Different STs in Spain, Greece, and Italy

In237 was present in ST78 (obtained in 2013) and ST90C (obtained in 2014) from the same institution in Greece. In916 was identified in ST78 (obtained in 2010) and ST114B (obtained in 2014) from the same institution in Italy. In624 was harbored in ST78, ST96, and ST108 from the same institution in Spain (all obtained in 2010). In*87* was detected in ST98, ST110, and ST141 from 2 different institutions in Greece (obtained in 2010 and 2014). In4873 was identified in ST114B from 2 different institutions in Greece (obtained in 2013) (Technical Appendix 2 Tables 1, 2). In110 with *bla*_VIM-1_ was present in ST105 from Croatia (obtained in 2013) and ST520 from Spain (obtained in 2012).

### Global Distribution of a Common NDM-1 Genetic Structure 

The most common genetic structure immediately surrounding the *bla*_NDMs_ (named NDM-GE-U.S.) in our collection was identical to that previously described on a 140.8 kb IncA/C plasmid (pNDM-U.S.; GenBank accession no. CP006661.1) found in *K. pneumoniae* ATCC BAA-2146 with *bla*_NDM-1_ (*24*). This bacterium was isolated in 2010 from the urine of a US hospital patient who had previously received medical care in India (*25*). NDM-GE-U.S., a 3,063-bp fragment consisting of ΔIS*Aba125*-*bla*_NDM-1_-*ble*_MBL_-*trpF*-*dsbC*, was present in 16/42 of NDM *E. cloacae* complex isolates among 14 different STs (88, 90B, 93, 114A, 279, 136, 182, 270, 435, 513, 524, 525, 609, and 832) obtained from Colombia, Romania, Philippines, Vietnam, South Africa, and Kenya (Technical Appendix 2 Table 3).

We determined the sequence similarity of the isolates with NDM-GE-U.S. to previously sequenced plasmids in the GenBank database. The similarity to pNDM-U.S. ranged from 7% to 81%, suggesting that different plasmids contained NDM-GE-U.S. Twelve of the isolates showed high similarity (range 93%–100%) to pK518_NDM1, a 106.8-kb IncFII plasmid with *bla*_NDM-1_ from China (GenBank accession no. CP023187). The remaining 4 isolates showed high similarity (range 98%–100%) with the 54-kb IncX3 plasmid pNDM-HN380 (n = 3) from China (*26*) and the 178.2-kb IncA/C plasmid p6234–178.193kb (n = 1) from the United States (GenBank accession no. CP010391).

## Discussion

The most common carbapenemase among *Enterobacter* spp. from our study was VIM, followed by NDM, KPC, OXA-48, and IMP. Carbapenemase-producing *Enterobacter* spp. was dominated by 2 global species, namely *E. xiangfangensis* with 1 major clone (ST114) and *E. hormaechei* subsp. *steigerwaltii* with 2 major clones (ST90 and ST93). ST114 and ST90 were divided into different clades; some of the clades (e.g., 90C and 114B) were located in certain geographic regions affiliated with specific carbapenemases, whereas other clades (114A and 90B) were distributed globally in association with different types of carbapenemases.

The taxonomy of *E. cloacae* complex is confusing, and uncertainty still remains about what species make up this complex. In the early 2000s, Hoffmann and Roggenkamp (*8*) sequenced *hsp60* and established 12 genetic clusters (I to XII) in *E. cloacae* complex. In 2005, the same authors further defined the taxonomy of *E. cloacae* complex and named cluster VII as *E. hormaechei* subsp. *hormaechei*, cluster VI as *E. hormaechei* subsp. *oharae*, and cluster VIII as *E. hormaechei* subsp. *steigerwaltii* (*27*). In 2014, Gu et al. (*28*) described a novel *Enterobacter* species obtained from sourdough in China named *E. xiangfangensis*, which clustered closest to *E. hormaechei*.

The first study that described the global distribution of *E. cloacae* clones was undertaken by Izedebski et al (*18*), who performed MLST on 173 cephalosporin-resistant *E. cloacae* isolates obtained from Israel and several countries in Europe. MLST identified 88 STs among this collection, with ST78, ST114, ST108, and ST66 being the most common and widespread clones. A ST78 isolate was positive for KPC-2, and a ST114 isolate was positive for VIM-1 (*18*). With the exception of this study from Izedebski et al (*18*), limited information is available regarding the global distribution of ST93, ST90, ST78, ST105, and ST108 and consists mainly of sporadic reports (*29*–*32*).

Chavda et al. (*9*) characterized 74 carbapenem-resistant *Enterobacter* spp. (more than half of the isolates were obtained from New Jersey, USA), and most possessed different *bla*_KPC_s, whereas only 2 isolates had *bla*_NDM-1_. *E*. *xiangfangensis* also dominated, and ST171 was the most common clone. ST171 was rare in our collection (n = 4) but did show genetic and geographic diversity. ST171 was divided into 3 clades: 171A, 171B, and 171C (Technical Appendix 2 Figure). Clades 171B and 171C are associated with *bla*_KPC_ from the United States and United Kingdom (Technical Appendix 2 Figure). Clade 171B (n = 2) contained *bla*_KPC-2_ from Colombia and *bla*_NDM-1_ from Guatemala. Clade 171A (n = 1) with *bla*_NDM-1_ was obtained from South Africa, and clade 171C with *bla*_KPC-3_ was obtained from the United States.

We noted interesting associations and geographic distribution between genetic structures surrounding carbapenemase genes and clades, clones, and species. First, identical genetic structures were situated in various STs within the same or different institutions of the same country (e.g., NDM-GE01 with *bla*_NDM-1_ in Vietnam; In87 and In237 with *bla*_VIM-1_ in Greece; In916 with *bla*_VIM-1_ in Italy; In624 with *bla*_VIM-1_ in Spain; and NDM-GE03 with *bla*_NDM-1_ in Guatemala). Second, identical genetic structure was present in different STs (ST105 and ST520), from different countries (e.g., In110 with *bla*_VIM-1_ in Croatia and Spain). Third, different genetic structures were present in the same STs and clades obtained from different countries (e.g., ST78 with In237 from Greece, ST78 with In916 from Italy, ST78 with In624 from Spain, ST114A with NDM-GE02 from Serbia, and ST114A with pNDM-U.S. from Romania). Last, an identical genetic structure (NDM-GE-U.S.) was found in different global species, STs, and clades.

These associations demonstrate that certain mobile genetic elements with carbapenemase genes have the ability to move between clones and clades of *Enterobacter* spp. on a global scale. This ability is highlighted by ST78 with *bla*_VIM-1_ within different integrons (In237, In916, and In624) that circulate between various countries (Greece, Italy, and Spain). As some STs are introduced into different countries, they apparently acquire the local genetic elements prevalent in that country. Of special concern is the description of a common NDM genetic structure, named NDM-GE-U.S., previously found on pNDM-U.S. and first described in a *K. pneumoniae* from the United States (*24*). NDM-GE-U.S. was present in different species, clones, and clades obtained from 6 countries spanning 4 continents. Sequence similarity analysis suggested that it was present on different types of plasmids (pK518_NDM1 and pNDM-HN380) among *Enterobacter* spp. with *bla*_NDM_.

Our results support the current understanding that the carbapenem resistance pandemic is the consequence of circulating clones and the spread of mobile genetic elements. We found that certain clones and clades (ST78, ST90C, ST96, ST114A, ST114C, and ST141) containing particular genetic structures (In87, In624, In916, In237, NDM-GE01, NDM-GE02, and NDM-GE03) and carbapenemases were circulating locally within the same or between different institutions in certain countries (Greece, Guatemala, Italy, Spain, Serbia, and Vietnam). Other global clones and clades (ST90B, ST93, and ST108) contained various genetic structures and carbapenemases.

A limitation of this study was that plasmids harboring carbapenemases were not reconstructed because of the limitations of short-read sequencing (*33*). The characterization of plasmids is vital to fully comprehend the molecular epidemiology of *Enterobacter* spp. with carbapenemases, and a follow-up study using long-read sequencing is currently under way. In the meantime, our study highlights the importance of surveillance programs using whole-genome sequencing to provide insight into the characteristics and global distribution of clones and clades as well as their association with mobile genetic elements surrounding the different carbapenemase genes.

Technical Appendix 1 Characteristics of *Enterobacter* spp. Isolates collected from the Merck Study for Monitoring Antimicrobial Resistance Trends, 2008–2014, and the AstraZeneca global surveillance program, 2012–2014.

Technical Appendix 2 Description of Merck Study for Monitoring Antimicrobial Resistance Trends and the AstraZeneca global surveillance program and characteristics of *Enterobacter* *aerogenes* and *E.* *cloacae* complex.
