# Supplementary material for: Genomic Epidemiology of Global Carbapenemase-Producing Enterobacter spp., 2008–2014
Source: Emerg Infect Dis. 2018 Jun;24(6):1010–9. doi: 10.3201/eid2406.171648 (PMC6004858; doi:10.3201/eid2406.171648)
Supplement: Technical Appendix 2 — Description of Merck Study for Monitoring Antimicrobial Resistance Trends and the AstraZeneca global surveillance program and characteristics of Enterobacter aerogenes and E. cloacae complex. [file 17-1648-Techapp-s2.pdf]

Article DOI: https://doi.org/10.3201/eid2405.171648

Genomic Epidemiology of Global Carbapenemase-Producing *Enterobacter* spp., 2008–2014

Technical Appendix 2

SMART and Astra-Zeneca Surveillance Programs

The Merck Study for Monitoring Antimicrobial Resistance Trends (SMART) program (2008–2014) included isolates from intra-abdominal and urinary tract infections from the following 55 countries: Egypt, Morocco, Kenya, South Africa, Tunisia (Africa); China, Hong Kong, India, Japan, Malaysia, Singapore, South Korea, Taiwan, Thailand, Vietnam (Asia); Croatia, Czech Republic, Estonia, France, Georgia, Greece, Germany, Hungary, Italy, Latvia, Lithuania, Portugal, Romania, Serbia, Slovenia, Spain, Switzerland, Turkey, United Kingdom (Europe); Argentina, Brazil, Chile, Colombia, Dominican Republic, Ecuador, Guatemala, Mexico, Puerto Rico, Panama, Uruguay, Venezuela (Latin America); Jordan, Lebanon, Israel, Saudi Arabia, UAE (Middle East); Canada, United States (North America); and Australia, New Zeeland, Philippines (South Pacific).

The AstraZeneca surveillance program of antimicrobial resistance (2012–2014) included isolates from intra-abdominal, urinary tract, blood, skin and soft tissue and lower respiratory tract infections from the following 42 countries: Egypt, Kenya, Nigeria, South Africa (Africa); China, South Korea, Taiwan, Thailand (Asia); Austria, Belgium, Bulgaria, Greece, Czech Republic, Denmark, France, Germany, Hungary, Italy, Macedonia, Portugal, Poland, Russia, Romania, Slovakia, Spain, Turkey, United Kingdom (Europe); Argentina, Brazil, Chile, Colombia, Mexico, Uruguay, Venezuela (Latin America); Lebanon, Israel, Syria, Kuwait (Middle East); United States (North America); and Australia, Philippines, Japan (South Pacific).

Both programs collected 100 consecutive clinically relevant non-repeat Gram-negative bacteria per annum from each institution.

Results Obtained with *Enterobacter aerogenes*

The phylogenetic tree containing *Enterobacter* spp. showed that *E. aerogenes* isolates were distant from other members of the *E. cloacae* complex (data not shown). A recent study showed that *E. aerogenes* was a closer relative to *Klebsiella pneumoniae* than the *E. cloacae* complex (*1*) and proposals to rename this species has been published (*2*).

The *E. aerogenes* (n = 10) from our study were obtained from intra-abdominal specimens (n = 4), urines (n = 5) and sputum (n = 1). The most common carbapenemase was KPC-2 from Brazil (n = 2), Colombia (n = 1), China (n = 1) and Germany (n = 1). This was followed by OXA-48 from Turkey (n = 2), Saudi Arabia (n = 1), Tunisia (n = 1) and NDM-6 from Guatemala (n = 1).

The bla_{KPC} was associated with the Tn*4401b* isoform (2 isolates from Brazil), $\text{NTE}_{\text{KPC-II}}$ identical to pECAZ159_2 [CP019006.1] (1 isolate from Colombia) and $\text{NTE}_{\text{KPC-Ib}}$ (2 isolates from China and Colombia). Isolates with $bla_{\text{OXA-48}}$ were part of Tn*1999.2*. The $bla_{\text{NDM-6}}$ from Guatemala had a unique genetic structural environment surrounding the carbapenemase named NDM-GE03 (MF327270).

β-lactamases, Antimicrobial Resistance Determinants, and Plasmid Analysis

Online Technical Appendix 1 Table 1 (https://wwwnc.cdc.gov/EID/article/24/6/17-1648-Techapp1.xlsx) contains the study number, GenBank accession no, species, date, country of isolation, ST/clade, β-lactamases, antimicrobial resistance determinants, plasmid replicon types and plasmid multilocus sequence typing (MLST). Most isolates contained different types of β-lactamases ranging from 1 – 6 per isolate; the most common non-carbapenemase was CTX-M-15 followed by OXA-1. Various additional antimicrobial resistance determinants were present ranging from 0–15 per isolate; these include different plasmid-mediated quinolone resistance determinants and aminoglycoside modifying enzymes (online Technical Appendix 1 Table 1).

As shown by Arrendo-Alonso and colleagues (*3*), our attempts at reconstructing plasmids using plasmidSPAdes, Recycler and cBar failed and these programs performed poorly using short read sequencing data. Plasmidfinder showed that the majority of isolates harbored several plasmid replicon types ranging from 0–5 per isolate and include the following: IncA/C2, IncHI2,

IncHI2A, IncFII, IncFIB, IncN, IncQ1, IncL/M, IncR, IncX3, IncP6, IncU, ColRNAI, IncQ1 and Col types. Unfortunately, only 22 carbapenemase-containing contigs also included a plasmid replicon or mob gene. The most common plasmid MLST was IncHI2: ST1. We identified 16 isolates that harbored a single β-lactamase (carbapenemase) associated with a single replicon type; those with only OXA-48 were positive for IncL/M, those with only KPC-2 for IncR and IncN, isolates with only VIM-1 for IncA/C and IncN, those with only NDM-1 were positive for IncR, and the NDM-7 for IncX3 (online Technical Appendix 1 Table 1).

References

1. Diene SM, Merhej V, Henry M, El Filali A, Roux V, Robert C, et al. The rhizome of the multidrug-resistant *Enterobacter aerogenes* genome reveals how new "killer bugs" are created because of a sympatric lifestyle. Mol Biol Evol. 2013;30:369–83. PubMed http://dx.doi.org/10.1093/molbev/mss236

2. Tindall BJ, Sutton G, Garrity GM. *Enterobacter aerogenes* Hormaeche and Edwards 1960 (Approved Lists 1980) and *Klebsiella mobilis* Bascomb et al. 1971 (Approved Lists 1980) share the same nomenclatural type (ATCC 13048) on the Approved Lists and are homotypic synonyms, with consequences for the name *Klebsiella mobilis* Bascomb et al. 1971 (Approved Lists 1980). Int J Syst Evol Microbiol. 2017;67:502–4. PubMed http://dx.doi.org/10.1099/ijsem.0.001572

3. Arredondo-Alonso S, Willems RJ, van Schaik W, Schürch AC. On the (im)possibility of reconstructing plasmids from whole-genome short-read sequencing data. Microb Genom. 2017;3:e000128. PubMed http://dx.doi.org/10.1099/mgen.0.000128

4. Hoffmann H, Roggenkamp A. Population genetics of the nomenspecies *Enterobacter cloacae.* Appl Environ Microbiol. 2003;69:5306–18. PubMed http://dx.doi.org/10.1128/AEM.69.9.5306-5318.2003

Technical Appendix 2 Table 1. The characteristics and global distribution of the different species among *Enterobacter cloacae* complex

Species (no.)	Hoffmann cluster	Global location (no.)	Carbapenemases (no.)	Dominant sequence types (no.)
Enterobacter xiangfangensis (n = 65)	VI (*4*)	Argentina (4), Australia (1), Brazil (2), Canada (1), Colombia (1), **Croatia (7),** Greece (4), Guatemala (2), India (1), Italy (1), Kuwait (2), Morocco (2), Nigeria (1), Philippines (4), **Romania (6)**, Russia (3), **Serbia (7),** South Africa (1), Spain (2), Taiwan (1), Tunisia (4), Turkey (3), USA (3), Venezuela (1), Vietnam (1)	IMP-13 (1), KPC-2 (10), KPC-3 (1), NDM-1 (21), OXA-48 (10), VIM-1 (15), VIM-1+OXA-48 (1), VIM-4 (2), VIM-4+OXA-48 (2), VIM-31+OXA-48 (2)	ST114 (19)
Enterobacter hormaechei subsp. steigerwaltii (n = 47)	VIII	Argentina (3), Australia (2), Austria (1), Belgium (1), Canada (1), China (1), Colombia (5), **Greece (11),** Hungary (1), Italy (2), Jordan (1), Mexico (1), Puerto Rico (1), Romania (2), Spain (1), Taiwan (1), Thailand (1), Turkey (1), USA (3), **Vietnam (7)**	IMP-4 (2), IMP-8 (2), IMP-14 (1), KPC-2 (10), KPC-2+NDM-1 (1), KPC-3 (1), KPC-3 (1), KPC-5 (1)<br>NDM-1 (7), NDM-1+OXA-48 (1), NDM-1+KPC-2 (1), OXA-48 (4), VIM-1 (15), VIM-4 (1), VIM-23 (1)	ST90 (10)<br>ST93 (14)
Enterobacter cloacae cluster III (n = 14)	III	Greece (3), Italy (1), Mexico (2), Philippines (1), Serbia (1), Spain (1), Taiwan (3), Turkey (2)	IMP-4 (1), IMP-8 (3), NDM-1 (1), OXA-48 (1), VIM-1 (5), VIM-5 (1), VIM-23 (2)	ST78 (10)
Enterobacter cloacae subsp. cloacae (n = 13)	XI	Colombia (2), Guatemala (1), Nigeria (1), Philippines (3), Puerto Rico (1), Tunisia (2), Vietnam 3)	KPC-2 (2), KPC-4 (1), NDM-1 (7), NDM-7 (1), OXA-48 (2)	Various
Enterobacter cloacae cluster IV (n = 9)	IV	Kenya (1), Kuwait (3), Philippines (1), Spain (1), USA (1), Venezuela (2)	KPC-2 (2), KPC-3 (1)<br>NDM-1 (1), NDM-7 (1)<br>OXA-48 (3), VIM-1 (1)	Various
Enterobacter hormaechei subsp. oharae (n = 6)	VI	Australia (1), China (2), Spain (2), Israel (1)	IMP-1 (2), IMP-4 (1)<br>OXA-48 (1), VIM-1 (2)	ST108 (6)
Enterobacter asburiae (n = 5)	I	Colombia (1), South Africa (1), USA (2), Taiwan (1)	IMP-8 (1), KPC-2 (3), NDM-1 (1)	Various
Enterobacter kobei (n = 1)	II	Spain (1)	VIM-1	ST520 (1)

Technical Appendix 2 Table 2. Characteristics and global distribution of major sequence types (≥10 isolates per ST) among *Enterobacter cloacae* complex*

Sequence Type (no.)	Clade (no.)	Carbapenemase and country of isolation (no.)	Genetic environment surrounding carbapenemase (GenBank accession no.)
E. cloacae cluster III	78 (10)	VIM-1 from Greece (3)	In237 (EF690695)
ST78 (n = 10)		VIM-1 from Italy (1)	In916 (KF856617)
		IMP-4 from the Philippines (1)	ND
		VIM-1 from Spain (1)	In624 (GQ422827)
		IMP-8 from Taiwan (3)	In73 (AF322577)
		OXA-48 from Turkey (1)	Tn*1999.2*
E. hormaechei subsp.	90B (3)	IMP-4 from Australia (1)	In809 (JX101693)
steigerwaltii ST90 (n = 10)	90C (7)	KPC-2 from Canada (1)	Tn*4401a*
		NDM from Romania (1)	pNDM-U.S. (CP006661.1)
		VIM-1 from Greece (7)	In237 (EF690695)
E. hormaechei subsp.	93 (14)	VIM-1 from Austria (1)	ND
steigerwaltii ST93 (n = 14)		OXA-48 form Belgium (1)	Tn*1999.2*
		IMP-8 from China (1)	In655 (HQ651093)
		NDM-1 from Romania (1)	pNDM-U.S. (CP006661.1)
		VIM-1 from Spain (1)	In3103 (LC169588)
		IMP-14 from Thailand (1)	In1314 (LC169569)
		KPC-2 from USA (1)	Tn*4401b*
		NDM-1 from Vietnam (5)	NDM-GE01 (MF288916)
		NDM-1+OXA-48 from Vietnam	NDM-GE01(MF288916)[NDM],Tn*1999.2*(OXA)
		(1)	Tn*1999.2*
		OXA-48 from Vietnam (1)	
E. xiangfangensis	114A (13)	VIM-4, OXA-48 from Kuwait (2)	In416 (LC169572) [VIM], Tn*1999.2* (OXA-48)
ST114 (n = 19)	114B (4)	OXA-48 from Morocco (1)	OXA-GE01 (MF327271)
	114C (2)	NDM-1 from Romania (1)	pNDM-U.S. (CP006661.1)
		NDM-1 from Serbia (7)	NDM-GE02 (MF346371)
		OXA-48 from Tunisia (2)	Tn*1999.2*, OXA-GE01 (MF327271)
		VIM-1 from Greece (3)	In4873 (LC169572)
		VIM-1 from Italy (1)	In916 (KF856617)
		VIM-1 from USA (1)	In1209 (LC169573)
		KPC-2 from USA (1)	Tn*4401b*

*The minor STs (5–9 isolates/ST) includes ST105 and ST108 (both with 6 isolates) were distinguished on the basis of their molecular epidemiology. ST105 from *E. xiangfangensis* was only present in Croatia where it contained *bla*$_{\text{VIM-1}}$ within In110; and in contrast, all the *E. hormaechei* subsp. *oharae* isolates belonged to ST108 and showed a global distribution (Australia [with *bla*$_{\text{IMP-4}}$], China [with *bla*$_{\text{IMP-1}}$], Israel [with *bla*$_{\text{OXA-48}}$], and Spain [with *bla*$_{\text{VIM-1}}$]). In, Integron; ND, not detected due to limitation of short read sequencing.

Technical Appendix 2 Table 3. Global distribution of sequence types, clades, genetic environments surrounding the carbapenemase genes among *Enterobacter cloacae* complex

Carbapenemase (no.)	Sequence types (no.)	Country of origin (no.)	Genetic environment (GenBank accession no.)
IMPs (14)	108 (2)	China (2)	ND
IMP-1 (2)	78A (1)	Philippines (1)	ND
IMP-4 (4)	90 (1)	Australia (1)	In809 (JX101693)
IMP-8 (6)	108 (1)	Australia (1)	ND
IMP-13 (1)	133 (1)	Australia (1)	ND
IMP-14 (1)	25 (1)	Taiwan (1)	In73 (AF322577)
	78A (3)	Taiwan (3)	In73 (AF322577)
	93 (1)	China (1)	In655 (HQ651093)
	204 (1)	Taiwan (1)	ND
	182 (1)	Spain (1)	In1319 (LC169585)
	93 (1)	Thailand (1)	In1314 (LC169569)
KPCs (33)	62 (2)	Argentina (2)	Tn*4401a*
KPC-2 (27)	66 (2)	Argentina (1), Brazil (1)	ND, Tn*4401b*
KPC-2+NDM-1 (1)	88 (2)	Colombia (2)	ND,Tn*4401b*
KPC-3 (3)	90B (1)	Canada (1)	Tn*4401a*
KPC-4 (1)	93 (1)	USA (1)	Tn*4401b*
KPC-5 (1)	110 (1)	Argentina (1)	ND
	114C (1)	USA (1)	Tn*4401b*
	171B (1)	Colombia (1)	KPC-GE01 (MF327266)
	200 (1)	Brazil (1)	Tn*4401b*
	418 (1)	Russia (1)	ND
	456 (1)	Colombia (1)	Tn*4401b*
	484 (2)	USA (2)	Tn*4401b*
	510 (2)	Colombia (2)	Tn*4401b*, KPC-GE02 (MF327263)
	511 (3)	Argentina (3)	ND
	516 (1)	Venezuela (1)	KPC-GE03 (MF327265)

Carbapenemase (no.)	Sequence types (no.)	Country of origin (no.)	Genetic environment (GenBank accession no.)
	523 (1)	Venezuela (1)	Tn*4401b*
	526 (1)	Venezuela (1)	Tn*4401b*
	535 (1)	USA (1)	Tn*4401b*
	610 (1)	Colombia (1)	NTEKPC-II identical pECAZ159_2 (CP019006.1)
	837 (1)	Colombia (1)	NTEKPC-II identical pECAZ159_2 (CP019006.1)
	88 (1)	Colombia	Tn*4401b*(KPC), pNDM-U.S. (CP006661.1)[NDM]
	171C (1)	USA (1)	Tn*4401d*
	461 (1)	USA (1)	Tn*4401b*
	515 (1)	USA (1)	Tn*4401d*
	137 (1)	Puerto Rico (1)	KPC-GE04 (MF351991)
	204 (1)	Puerto Rico (1)	Tn*4401b*
NDMs (41)	66 (1)	Canada (1)	NDM-GE-VF‡
NDM-1 (38)	90B (1)	Romania (1)	NDM-GE-U.S.†
NDM-1+OXA-48 (1)	93 (1)	Romania (1)	NDM-GE-U.S.†
NDM-7 (2)	93 (6)	Vietnam (6)	NDM-GE01 (MF288916)
	114A (1)	Romania (1)	NDM-GE-U.S.†
	114A (7)	Serbia (7)	NDM-GE02 (MF346371)
	136 (2)	Philippines (2)	NDM-GE-U.S.†
	171 (2)	South Africa (1) Guatemala(1)	GE-pC06114_1§
	182 (3)	Guatemala (1)	NDM-GE03 (MF327270)
	270 (1)	Philippines (1)	NDM-GE03 (MF327270)
	279 (3)	Vietnam (1)	NDM-GE-U.S.†
	418 (1)	Philippines(1)	NDM-GE01 (MF288916)
	435 (1)	Romania (3)	NDM-GE-U.S.†
	462 (1)	India (1)	NDM-GE-U.S.†
	513 (1)	South Africa (1)	GE-PEL¶
	518 (1)	Vietnam (1)	NDM-GE-U.S.†
	519 (1)	Vietnam (1)	NDM-GE01 (MF288916)
	524 (1)	Serbia (1)	NDM-GE-U.S.†
	525 (1)	Guatemala (1)	NDM-GE04 (MF327264)
	609 (1)	Vietnam (1)	NDM-GE03 (MF327270)
	832 (1)	Philippines (1)	NDM-GE01 (MF288916)
	836 (1)	Philippines (1)	NDM-GE-U.S.†
	93 (1)	Kenya (1)	NDM-GE-U.S.†
	834 (1) 835 (1)	Nigeria (1)	NDM-GE-U.S.†
		Vietnam (1)	GE-PittNDM01#
		Philippines (1)	NDM-GE01(MF288916)[NDM], Tn1999.2(OXA)
		Philippines (1)	GE-pEC50-NDM7**
			GE-pEC50-NDM7**
OXA-48 (21)	78A (1)	Turkey (1)	ND
	93 (2)	Belgium (1), Vietnam (1)	ND
	98 (1)	Russia (1)	ND
	108 (1)	Israel (1)	ND
	109 (1)	Russia (1)	ND
	114A (3)	Morocco (1)	OXA-GE01 (MF327271)
	121 (2)	Tunisia (2)	OXA-GE01 (MF327271), Tn*1999.2*
	182 (1)	Morocco (1)	OXA-GE01 (MF327271)
	190 (1)	Nigeria (1)	ND
	200 (1)	Tunisia (1)	Tn*1999.2*
	433 (2)	Turkey (1)	OXA-GE02 (MF327268)
	435 (1)	Turkey (1)	OXA-GE03 (MF327269)
	456 (2)	Kuwait (2)	OXA-GE04 (MF327267)
	521 (1)	Kuwait (1)	ND
	522 (1)	Tunisia (2)	Tn*1999.2*
		Jordan (1)	Tn*1999.2*
		Spain (1)	Tn*1999.2*
VIMs (51)	66 (1)	Taiwan (1)	ND
VIM-1 (39)	78 (5)	Greece (3)	In237 (EF690695)
VIM-1+OXA-48 (1)	88 (1)	Italy (1)	In916 (KF856617)
VIM-4 (3)	90C (7)	Spain (1)	In624 (GQ422827)
VIM-4+OXA-48 (2)	93 (2)	Italy (1)	In1318 (LC169584)
VIM-5 (1)	96 (1)	Greece (7)	In237 (EF690695)
VIM-23 (3)	98 (1)	Austria (1)	ND
VIM-31+OXA-48 (2)	105 (6)	Spain (1)	In3103 (LC169588)
	108 (2)	Spain (1)	In624 (GQ422827)
	110 (2)	Greece (1)	In87 (AY648125)
	114B (4)	Croatia (6)	In110 (AJ969234)
	114C (1)	Spain (2)	In624 (GQ422827)

Carbapenemase (no.)	Sequence types (no.)	Country of origin (no.)	Genetic environment (GenBank accession no.)
	136 (1)	Greece (2)	In87 (AY648125)
	141 (2)	Greece (3)	In4873 (LC169572)
	514 (1)	Italy (1)	In916 (KF856617)
	520 (1)	USA (1)	In1209 (LC169573)
	527 (1)	Tunisia (1)	In1315 (LC169570)
	418 (1)	Greece (2)	In87 (AY648125)
	62 (1)	Italy (1)	ND
	265 (2)	Spain (1)	In110 (AJ969234)
	114A (2)	Austria (1)	In1373 (LC224311)
	512 (1)	Croatia (1)	ND (VIM), Tn*1999.2* (OXA)
	175 (1)	Hungary (1)	ND
	796 (2)	Romania (2)	In1323 (LC169579)
	200 (2)	Kuwait (2)	In416 (AJ704863), ND (OXA)
		Turkey (1)	In1316 (LC169578)
		Mexico (1)	In1372 (LC224310)
		Mexico (2)	In1374 (LC224312)
		Turkey (2)	In669 (JN982330)[VIM], OXA-GE03 (MF327269)[OXA]

*In, Integron; ND, not detected due to limitation of short read sequencing.
†Identical to a region on pNDM-U.S. (CP006661.1).
‡Identical to a region on pNDM-VF (KR733543.1).
§Identical to a region on pC06114_1 (CP016035.1).
¶Similar to a region on *P. mirabilis* PEL (KF856624.1).
#Identical to a region on PittNDM01 plasmid1 (CP006799.1).
**Identical to a region pEC$_{50}$-NDM7 (KX470735.1).

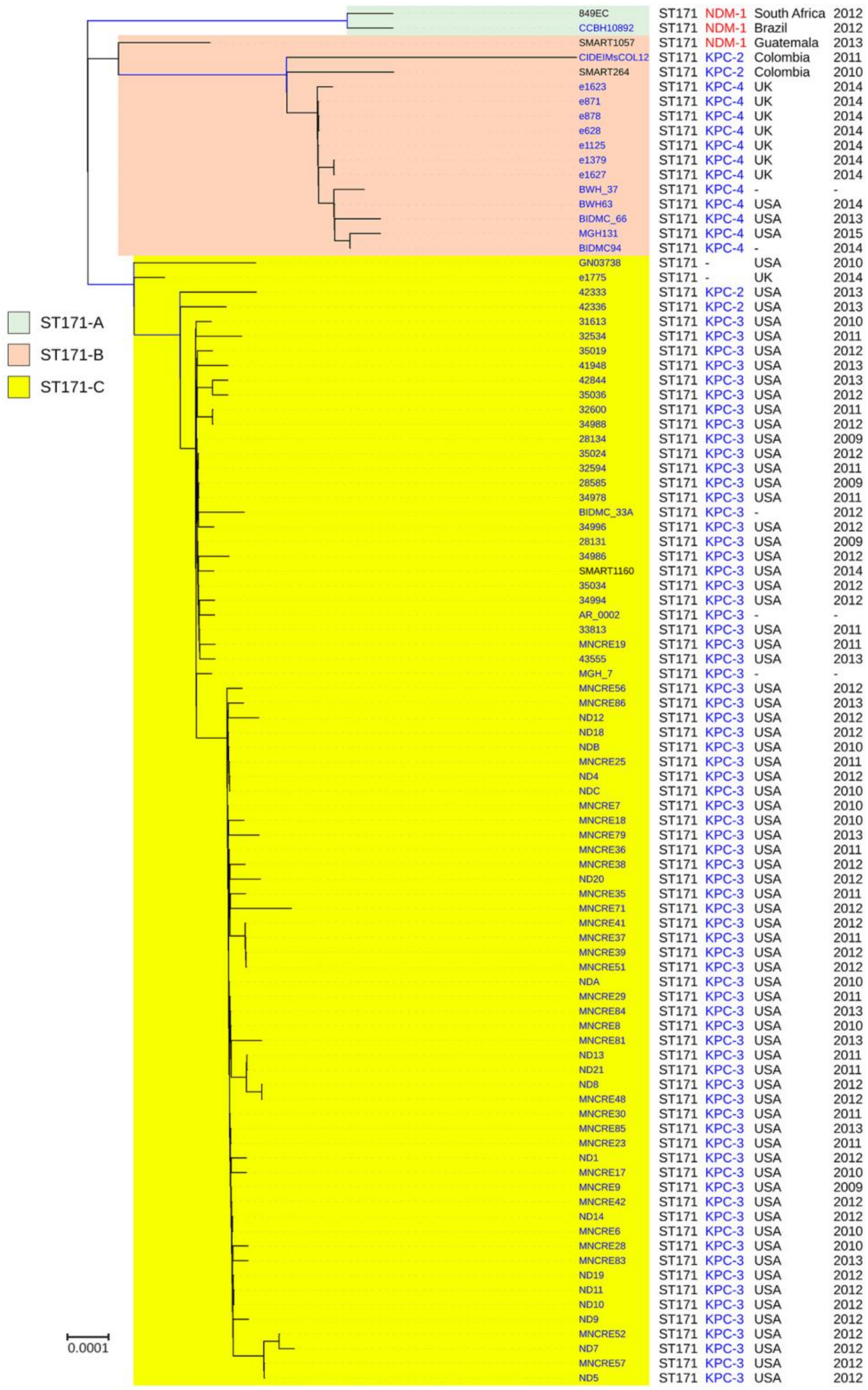

Technical Appendix 2 Figure. Phylogenetic tree of the different clades among *Enterobacter xiangfangensis* ST171. Number of isolates: 338. Root: *Enterobacter hormaechei* subsp. *hormaechei* ATCC49162. Number of total core SNP found: 317867. Number of core SNP used to draw the tree: 27,705 (after phages and recombination sites were excluded).
